# O-GlcNAcylation of G6PD promotes the pentose phosphate pathway and tumor growth

**DOI:** 10.1038/ncomms9468

**Published:** 2015-09-24

**Authors:** Xiongjian Rao, Xiaotao Duan, Weimin Mao, Xuexia Li, Zhonghua Li, Qian Li, Zhiguo Zheng, Haimiao Xu, Min Chen, Peng G. Wang, Yingjie Wang, Binghui Shen, Wen Yi

**Affiliations:** 1 Institute of Biochemistry, College of Life Sciences, Zhejiang University, Hangzhou 310058, China; 2 Collaborative Innovation Center for Diagnosis and Treatment of Infectious Diseases, Hangzhou 310003, China; 3 State Key Laboratory of Toxicology and Medical Countermeasures, Beijing Institute of Pharmacology and Toxicology, Beijing 100850, China; 4 Zhejiang Cancer Hospital, Zhejiang Cancer Research Institute, Hangzhou 310022, China; 5 School of Life Science and the State Key Laboratory of Microbial Technology, National Glycoengineering Research Center, Shandong University, Shandong 250100, China; 6 Department of Chemistry, Georgia State University, Atlanta, Georgia 30303, USA; 7 State Key Laboratory for Diagnosis and Treatment of Infectious Diseases, The First Affiliated Hospital of Zhejiang University, Hangzhou 310003, China; 8 Department of Radiation Biology, City of Hope National Medical Center, Duarte, California 91010, USA

## Abstract

The pentose phosphate pathway (PPP) plays a critical role in macromolecule biosynthesis and maintaining cellular redox homoeostasis in rapidly proliferating cells. Upregulation of the PPP has been shown in several types of cancer. However, how the PPP is regulated to confer a selective growth advantage on cancer cells is not well understood. Here we show that glucose-6-phosphate dehydrogenase (G6PD), the rate-limiting enzyme of the PPP, is dynamically modified with an O-linked β-N-acetylglucosamine sugar in response to hypoxia. Glycosylation activates G6PD activity and increases glucose flux through the PPP, thereby providing precursors for nucleotide and lipid biosynthesis, and reducing equivalents for antioxidant defense. Blocking glycosylation of G6PD reduces cancer cell proliferation *in vitro* and impairs tumor growth *in vivo*. Importantly, G6PD glycosylation is increased in human lung cancers. Our findings reveal a mechanistic understanding of how O-glycosylation directly regulates the PPP to confer a selective growth advantage to tumours.

## Abstract (web summary)

The pentose phosphate pathway is aberrantly activated in cancer cells but the mechanism is unclear. Here, the authors show that G6PD, the rate-limiting enzyme in the pathway, is post-translationally modified with a sugar moiety under hypoxic conditions leading to increased production of precursors for macromolecular synthesis and antioxidants.

## Graphical abstract

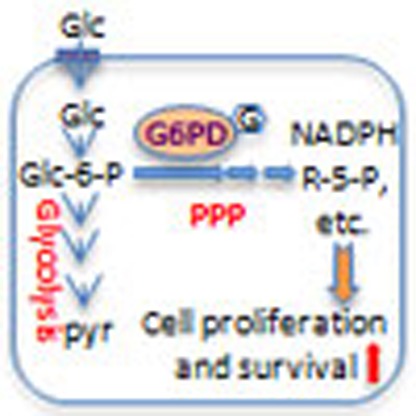

Altered metabolism is a key characteristic of cancer cells. The metabolic flux in cancer cells is markedly reprogrammed to provide elevated amounts of building blocks for anabolic biosynthesis of macromolecules during rapid cell growth and proliferation^1^,^2^. In addition, cancer cells often possess enhanced capability to regulate redox homoeostasis to protect against oxidative stress in the tumor microenvironment^3^,^4^. However, the molecular mechanisms by which cancer cells sense metabolic signal to couple anabolic synthesis and redox homoeostasis to promote cancer cell proliferation and cell survival are not well understood.

Glucose flux through the glycolytic pathway can be diverted into the pentose phosphate pathway (PPP). The PPP plays a vital role in meeting the cellular demands for anabolic biosynthesis and providing anti-oxidative defense^5^. It generates ribose-6-phosphate for *de novo* synthesis of DNA and RNA, and the reducing equivalent reduced NADPH for reductive biosynthesis of lipids. NAPDH also functions as an important antioxidant for detoxification of high levels of reactive oxygen species (ROS) produced during rapid cell proliferation to promote cell survival. Activity of the PPP is known to be upregulated in cancer cells compared with normal epithelial cells^6^. Knocking down of key enzymes in the PPP inhibits tumor growth and sensitizes cancer cells to oxidative stress^7^,^8^. Glucose-6-phosphate dehydrogenase (G6PD) catalyses the first committed and rate-limiting step of the PPP. It catalyses the oxidation of G6P to 6-phosphogluconate and produces NADPH in the presence of NADP^+^. G6PD is considered the pacesetter of the PPP and the primary control point for NADPH production. G6PD activity is subjected to various regulatory mechanisms ranging from transcription to translation, further illustrating its importance in regulating cellular metabolism^5^.

O-linked β-N-acetylglucosamine (O-GlcNAc) is a dynamic and inducible post-translational modification of serine and/or threonine residues of nuclear and cytosolic proteins^9^. In cells, a single set of antagonistic enzymes—O-GlcNAc transferase (OGT) and O-GlcNAc hydrolase are responsible for the addition and removal of GlcNAc moiety, respectively. O-GlcNAcylation has been identified in numerous proteins and shows a complex crosstalk with protein phosphorylation^10^. Increasing evidence has shown that O-GlcNAcylation serves important roles in regulating gene transcription, cellular signalling and stress responses^11^. Generally considered as a ‘nutrient sensor' of cells, recent findings also indicate that O-GlcNAcylation may actively and directly participate in regulating cellular metabolism^12^. O-GlcNAc levels are significantly elevated in various cancers. Aberrant O-GlcNAcylation has been shown to correlate with cancer cell proliferation, invasion and metastasis both *in vitro* and *in vivo*^13^,^14^,^15^. Thus, deregulation of O-GlcNAcylation appears to be a general feature of cancer cells. However, the detailed mechanisms by which protein-specific O-GlcNAcylation contributes to cancer metabolic reprogramming and tumorigenesis remain largely unknown.

Here, we present evidence that O-GlcNAcylation of G6PD coordinates cancer cell anabolic biosynthesis and redox homoeostasis to promote tumor growth *in vivo*. Mechanistically, glycosylation activates G6PD activity and increases glucose flux through the PPP, thereby generating precursors for nucleotide and lipid biosynthesis, and reducing equivalents for antioxidant defense. In addition, glycosylation of G6PD promotes cancer cell proliferation *in vitro* and tumor growth *in vivo*. Importantly, G6PD glycosylation is elevated in human lung cancers. Thus, our study identifies a previously unknown mechanism for the regulation of cancer metabolism and tumor growth by protein O-glycosylation.

## Results

### G6PD is dynamically modified by O-GlcNAc at serine 84

**Figure 1:**
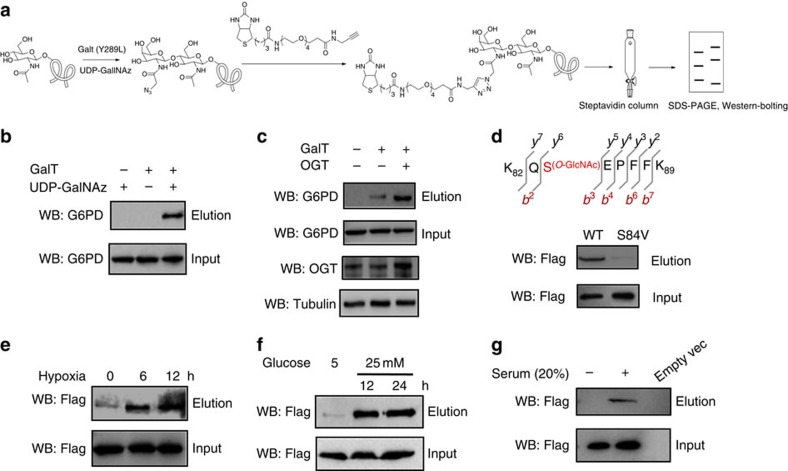
G6PD is dynamically modified by O-GlcNAc at serine 84. (**a**) Chemoenzymatic labelling approach for biotinylation, capture and detection of O-GlcNAcylated G6PD from cells. Endogenous O-GlcNAcylated proteins in cell lysates were chemoenzymatically tagged with an azido-galactose sugar using a mutant galactosyltransferase (GalT, Y289L) and the non-natural nucleotide sugar analogue UDP-GalNAz, and then biotinylated by reaction of the azido-galactose sugar with an alkyne-functionalized biotin molecule. The biotinylated proteins were pulled down using streptavidin beads, and eluted with SDS. Lysates before pull down (input) and the captured proteins (elution) were immunoblotted with an antibody towards G6PD. (**b**) Detection of O-GlcNAcylated G6PD levels from 293T cells. Lysates prior to pull down (input) and the captured proteins (elution) were immunoblotted with an antibody towards G6PD. Control experiments in the absence of GalT or UDP-GalNAz demonstrated selective labelling of the O-GlcNAcylation on G6PD. (**c**) Detection of O-GlcNAcylated G6PD levels from 293T cells overexpressing OGT using the chemo-enzymatic method. (**d**) Peptide sequence and glycosylation site identified by LTQ-Orbitrap MS/MS. Glycosylation levels of WT G6PD compared with the S84V mutant expressed in 293T cells as determined by the chemoenzymatic method. (**e**) Determination of O-GlcNAcylation levels of G6PD under hypoxic treatment for indicated periods of time by the chemoenzymatic method. (**f**) Detection of O-GlcNAcylation levels of G6PD cultured in media with different glucose concentrations by the chemo-enzymatic method. (**g**) Detection of O-GlcNAcylation levels of G6PD in A549 cells on serum stimulation by the chemoenzymatic method.

Previous proteomic studies^16^,^17^ have revealed that a large number of metabolic enzymes including G6PD are modified by O-GlcNAc in eukaryotic cells, suggesting a critical role of O-GlcNAcylation in regulating cell metabolism. To investigate whether O-GlcNAcylation directly regulates the PPP, we focus on the rate-limiting enzyme G6PD. To confirm that G6PD is O-GlcNAcylated, we employed a well-established chemo-enzymatic labelling approach^16^. We enzymatically labelled all O-GlcNAc-modified proteins from 293T cell lysates with an azido-N-acetylgalactosamine sugar. Labelled proteins were then biotinylated via Cu(I)-mediated [3+2] azide-alkyne cycloaddition (CuACC) chemistry and captured with streptavidin-agarose beads. Subsequent immunoblotting of the captured proteins with an antibody against G6PD showed strong O-GlcNAcylation of G6PD (Fig. 1a,b). Calculation of the glycosylated protein versus total G6PD protein yielded an estimate of basal glycosylation level to be 8±2.1%. Glycosylation of G6PD was enhanced by 4.5-fold in cells by overexpression of OGT, the enzyme responsible for the addition of O-GlcNAc onto proteins (Fig. 1c). To corroborate with this result, we immunoprecipitated endogenous G6PD from 293T cell lysates and immunoblotted with a pan-anti-O-GlcNAc antibody (CTD110.6), which yielded a distinct signal albeit much lower sensitivity compared to the chemo-enzymatic approach (Supplementary Fig. 1). Thus, G6PD is O-GlcNAc-modified in cells.

To identify the site(s) of O-GlcNAcylation on G6PD, we transiently co-expressed Flag-tagged G6PD and OGT in 293T cells. After immunoprecipitation and in-gel trypsin digestion of G6PD, peptides were subjected to high-resolution mass spectrometry analysis (nanoLC-LTQ-CID/ETD-Orbitrap). We identified a single site of O-GlcNAcylation at Serine 84, a highly conserved residue in homologues proteins among mammals (Fig. 1d and Supplementary Fig. 2). Mutation of Ser84 to valine abolished the glycosylation signal, supporting that Ser84 is the only site of O-GlcNAcylation on G6PD (Fig. 1d). Notably, no other forms of modification such as phosphorylation were detected at the same serine residue.

Accumulating research data have suggested that protein O-GlcNAcylation is induced in response to various forms of cell stress^18^, and that elevation of O-GlcNAcylation plays an important role in modulating critical biological pathways to improve cell survival^18^. To investigate whether O-GlcNAcylation of G6PD is dynamically regulated in cells, we subjected cells to different stress conditions and probed the O-GlcNAcylation levels of G6PD. Notably, G6PD glycosylation was induced in a time-dependent manner under hypoxic conditions (3.1- to 4.7-fold induction, Fig. 1e). We found that hypoxic treatment induced global O-GlcNAc levels in cells and endogenous OGT expression (Supplementary Fig. 3A). Consistently, *in vitro* enzymatic assays demonstrated that OGT activity in cell lysates was increased under hypoxia (Supplementary Fig. 3B). Hypoxia is also known to induce profound changes in glucose metabolism, including increasing glucose uptake via the transcriptional upregulation of glucose transporters^19^. Indeed, the hypoxic treatment significantly enhanced glucose uptake rate in our study (Supplementary Fig. 3C), nicely correlated with the induction of O-GlcNAcylation level. In addition, inhibition of glucose uptake by a small-molecule inhibitor suppressed the induction of G6PD O-GlcNAcylation under hypoxia (Supplementary Fig. 3D). Thus, hypoxia induces G6PD glycosylation likely by increasing OGT expression and cellular glucose concentration, which serves as a biosynthetic precursor for O-GlcNAc. Similarly, G6PD glycosylation was also stimulated when cells were treated with high glucose concentration (4.2- to 5.8-fold induction), or with serum (1.7-fold induction; Fig. 1f,g). In agreement with previous reports^3^, growth factor (serum) stimulation significantly induced cellular glucose uptake rate in our study (Supplementary Fig. 3E). Taken together, these results demonstrate that G6PD O-GlcNAcylation is dynamically regulated in response to different cellular conditions, suggesting a signalling role of G6PD glycosylation in cells.

### O-GlcNAcylation of G6PD activates enzyme activity

**Figure 2:**
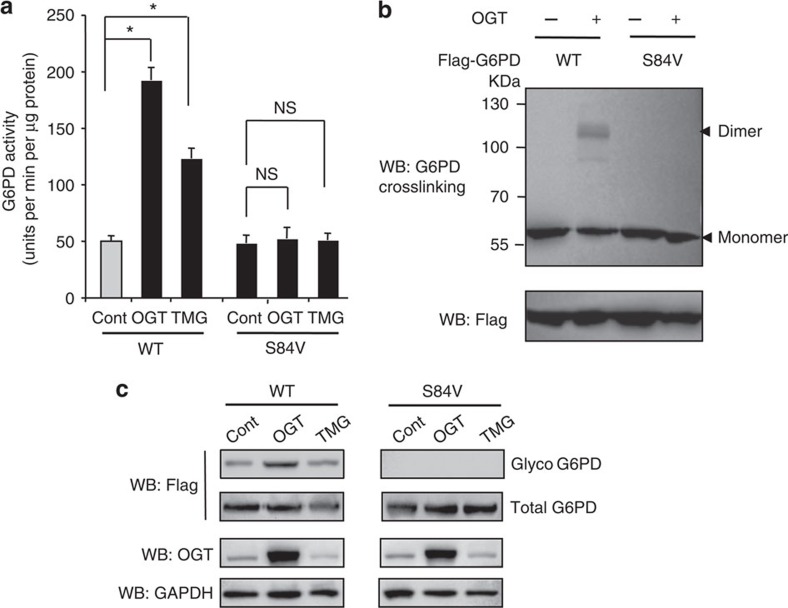
O-GlcNAcylation regulates G6PD enzyme activity and oligomerization. (**a**) Enzymatic activities of WT and S84V G6PD purified from untreated (Cont), OGT-overexpressing, and thiamet-G (TMG) treated 293T cells (*n*=4 experiments). Error bars denote the standard deviation of the mean (mean±s.d.). Statistical analysis was performed by one-way analysis of variance and Bonferroni comparison post-test (*P<0.05). (**b**) Oligomerization state of Flag-tagged WT and S84V G6PD in cells. Crosslinking with glutaraldehyde and followed by immunoblotting detected dimeric and monomeric G6PD. Flag-tagged G6PD protein input was shown in the lower panel. (**c**) Immunoblots of G6PD glycosylation under different cellular treatments. NS, not significant.

To understand the biological significance of G6PD O-GlcNAcylation, we first examined the effect of O-GlcNAcylation on G6PD enzyme activity. Enhancing O-GlcNAcylation in 293T cells by OGT overexpression or pharmacological inhibition of O-GlcNAc hydrolase with a specific inhibitor thiamet-G^20^ significantly increased G6PD enzyme activity by two to fourfold (Fig. 2a). Mutation of S84 to valine (S84V) retained a similar activity as compared to the wild-type (WT) G6PD. However, the S84V mutant showed negligible response in enzyme activity on OGT overexpression or thiamet-G treatment (Fig. 2a). Similar results were obtained when cells were subjected to hypoxic treatments to induce G6PD glycosylation (Supplementary Fig. 4). Thus, these results suggest that S84 is an important regulatory site of G6PD activity.

To further understand the effect of S84 glycosylation on G6PD activity, we examined the steady-state kinetics of G6PD with different glycosylation levels. Specifically, we compared the kinetics of Flag-tagged WT G6PD expressed in 293T cells in the presence or absence of OGT overexpression. Flag-tagged G6PD with high O-GlcNAcylation displayed substantially higher catalytic efficiency (2.9-fold increase in *k*_cat_/*K*_m_ for NADP+ and 1.4-fold increase for G6P) than Flag-tagged G6PD with low O-GlcNAcylation (Supplementary Table 1). Notably, the *K*_m_ for NADP^+^ showed about twofold decrease in G6PD with high glycosylation levels, indicating a higher binding affinity of NADP^+^ to the enzyme. Consistently, the calculated dissociation constant (*k*_d_) for NADP^+^ using the fluorescence titration assay was 2.6 μM for G6PD with high glycosylation levels, which is about threefold lower than G6PD with low glycosylation levels (7.3 μM). Thus, the kinetics study suggests that O-GlcNAcylation induces G6PD activity likely by enhancing the binding affinity of NADP^+^ to G6PD.

The G6PD protein exists in different oligomeric states, ranging from monomer, dimer, tetramer to hexamer. Studies have shown that only dimeric and tetrameric forms of G6PD are catalytically active^21^,^22^. To examine whether S84 glycosylation affects oligomerization states of G6PD, we performed a protein crosslinking experiment using glutaraldehyde. As shown in Fig. 2b, enhancing O-GlcNAcylation levels by OGT overexpression resulted in substantial increased formation of dimeric G6PD, but not S84V mutant. Control immunoblots were shown in Fig. 2c. These results suggest that O-GlcNAcylation activates G6PD in part by perturbing the equilibrium between different oligomeric forms to favour a higher oligomeric state.

### O-GlcNAcylation of G6PD promotes the PPP

**Figure 3:**
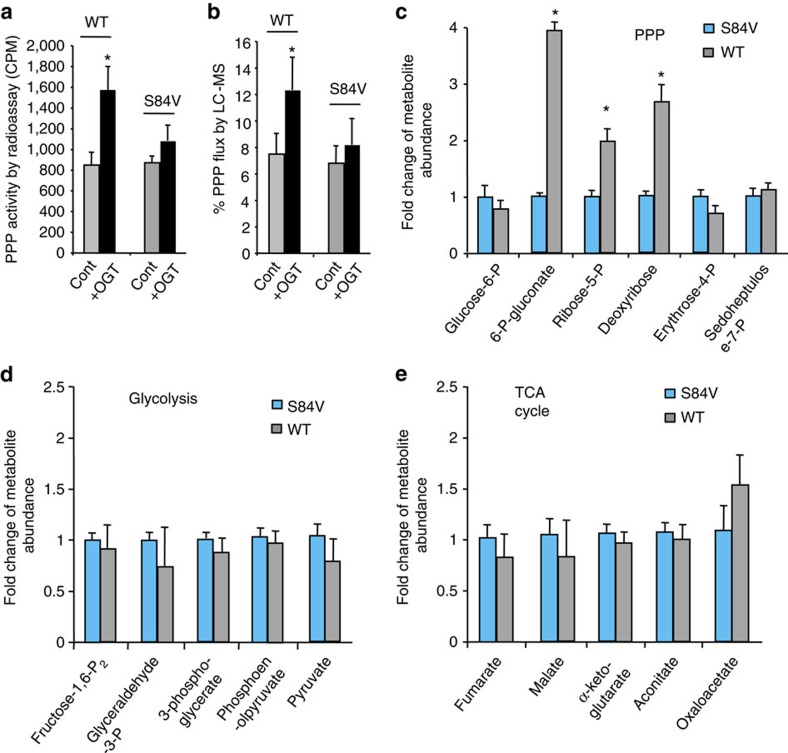
O-GlcNAcylation of G6PD regulates metabolic flux through the PPP. (**a**) PPP activity in WT and S84V G6PD replacement cells in the absence or presence of OGT overexpression, as determined by the amount of released ^14^CO_2_ from [1-^14^C]-glucose (*n*=3 assays). (**b**) Percentage of glucose flux through the PPP in WT and S84V G6PD replacement cells in the absence or presence of OGT overexpression, as measured by the relative accumulation of singly ^13^C-labelled lactate from cells metabolically labelled with 1,2-^13^C-glucose using reverse-phase triple-quadrupole LC–MS (*n*=3 assays). (**c**–**e**) Targeted analysis of abundance of different metabolites in major glucose metabolic pathways: PPP (**c**), glycolysis (**d**), and TCA cycle (**e**) in WT and S84V G6PD replacement A549 cells on OGT overexpression (*n*=3 experiments). Error bars denote mean±s.d. Statistical analysis was performed by Student's *t*-test (*P<0.05).

To create a system to study the effect of G6PD S84 glycosylation on cellular metabolism, we depleted endogenous G6PD and stably expressed small hairpin RNA (shRNA)-resistant Flag-tagged WT or S84V G6PD in A549 lung cancer cells (henceforth referred to as WT G6PD or S84V G6PD replacement cells, Supplementary Fig. 5). As the first and rate-limiting step of the PPP, induction of G6PD activity by O-GlcNAcylation is expected to have an impact on the metabolic flux of the PPP. Indeed, we observed a significant increase in flux (∼2.1-fold) through the oxidative PPP, as measured by the amount of released ^14^CO_2_ from [1-^14^C]-glucose, when OGT was overexpressed in WT G6PD replacement cells. In contrast, in S84V G6PD replacement cells, PPP flux exhibited a modest increase on OGT overexpression (Fig. 3a). The small increase in PPP flux is likely due to the inhibition of phosphofructokinase 1 (PFK1), a key regulatory enzyme in glycolysis pathway, by O-GlcNAcylation as shown in the previous study^23^. Nevertheless, this result demonstrates that S84 O-GlcNAcylation of G6PD induced glucose flux through the PPP. This observation was independently verified by a metabolic tracing experiment, in which cells were metabolically labelled with 1,2-^13^C-glucose and the relative isotopic enrichment accumulation of singly versus doubly ^13^C-labelled lactate was measured by liquid chromatography–mass spectrometry (LC–MS) analysis (Fig. 3b). In addition, we showed that hypoxia induced similar upregulation in PPP flux and that the upregulation was dependent on G6PD glycosylation (Supplementary Fig. 6).

Our previous investigation on O-GlcNAcylation of PFK1 has demonstrated that O-GlcNAcylation inhibits PFK1 enzyme activity, resulting in rerouting a portion of glucose flux through the PPP^23^. We then carried out experiments to further understand the differential contribution to PPP regulation by glycosylation of PFK1 and G6PD. PFK1 has three isoforms (PFKL, PFKP and PFKM) that show different regulatory response by O-GlcNAcylation with PFKM being the least responsive^23^. We generated A549 stable cell lines that harbour only single isoforms. In addition, these PFK1 isoforms were mutated to become glycosylation-deficient (referred to as PFKL-SA, PFKP-SA and PFKM-SA). Consistent with the previous study, PPP flux increased significantly in cells expressing WT PFK1 isoforms on OGT overexpression (Supplementary Fig. 7). The expression of glycosylation-deficient mutants of PFK1 (PFKL-SA or PFKP-SA) resulted in a large suppression of PPP flux, but the expression of PFKM-SA mutant only resulted in a modest decrease in PPP flux (Supplementary Fig. 7). Replacement of WT G6PD with S84V G6PD further suppressed PPP flux in all three cases. These results suggest that O-GlcNAcylation regulates the PPP activity through the coordinated action of PFK1 and G6PD. In cases where PFKL or PFKP expression is predominant, PFK1 glycosylation exerts more control in regulating the PPP flux. On the other hand, when PFKM expression is predominant, G6PD glycosylation plays a more dominant role in PPP regulation.

To further examine the impact of G6PD glycosylation on major metabolic pathways, we subjected cell extracts to LC–MS analysis to determine the relative enrichment of specific metabolites. On OGT overexpression, the metabolic profile of WT G6PD replacement cells demonstrated a general increase in PPP metabolites, as compared with S84V G6PD replacement cells (Fig. 3c). Metabolites involved in glycolytic pathway and tricarboxylic acid cycle showed no significant difference between WT and S84V G6PD replacement cells (Fig. 3d,e). Control experiments were performed in parental A549 cells on G6PD overexpression (Supplementary Table 2). The metabolite patterns suggested that S84 glycosylation imparted a specific metabolic effect on glucose metabolism through the PPP.

### G6PD glycosylation promotes cell proliferation and survival

**Figure 4:**
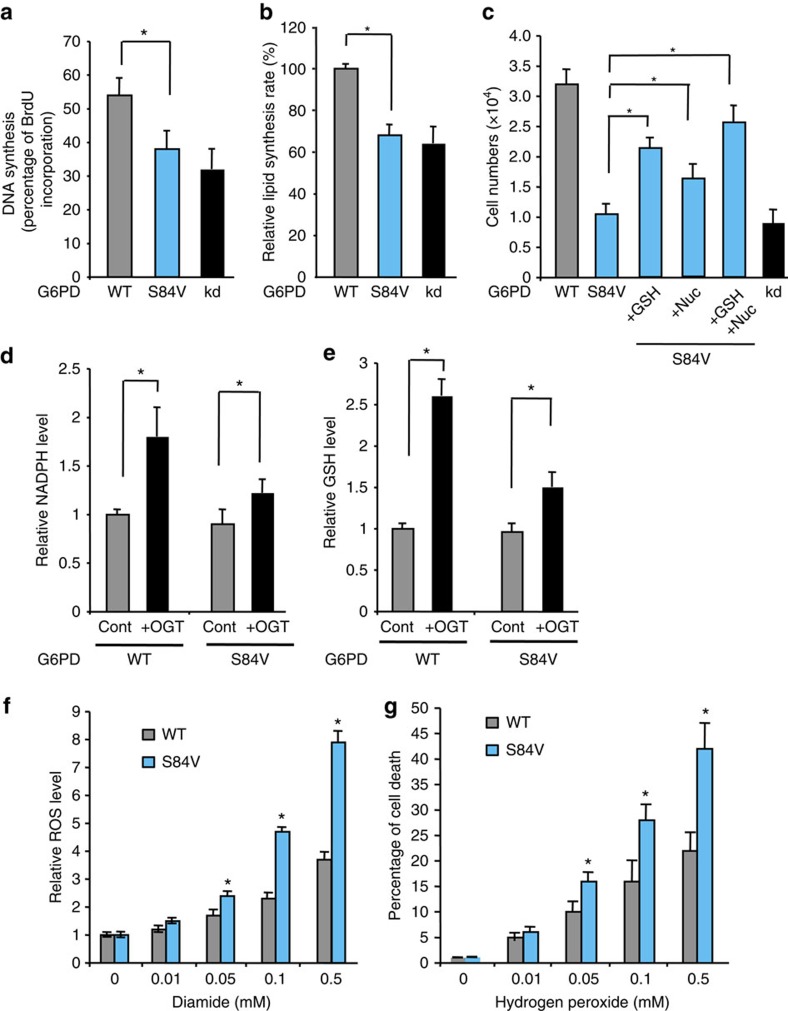
O-GlcNAcylation of G6PD promotes cellular biosynthesis, cell proliferation and antioxidant defense. (**a**) DNA synthesis in WT and S84V G6PD replacement A549 cells under hypoxic conditions, as determined by 5-bromo-2'-deoxyuridine incorporation assays. Control experiment was performed in G6PD depleted A549 cells (*n*=4 assays). (**b**) Lipogenesis in WT and S84V G6PD replacement A549 cells under hypoxic conditions, as determined by ^14^C-glucose labelled lipid incorporation assays. Control experiment was performed in G6PD depleted cells (*n*=3 assays). (**c**) Cell proliferation rates under hypoxic conditions of WT and S84V G6PD replacement A549 cells. Rescue experiments were carried out by the addition of GSH (5 mM), Nuc (four ribonucleotides and four deoxyribonucleotides), or both in the culture medium. Control experiments were performed in G6PD depleted A549 cells (*n*=3 assays). Cell numbers were determined by trypan blue counting. (**d**) NADPH and (**e**) GSH levels in WT and S84V G6PD replacement A549 cells in the absence or presence of OGT overexpression under normoxic conditions (*n*=3 assays). (**f**) Cellular reactive oxygen species (ROS) levels induced by different concentrations of diamide in WT and S84V G6PD replacement A549 cells for 2 h under normoxic conditions (*n*=4 assays). (**g**) Percentage of cell death induced by varying hydrogen peroxide concentrations in WT and S84V G6PD replacement A549 cells for 1 h under normoxic conditions (*n*=4 assays). Error bars denote mean±s.d. of three independent experiments. Statistical analysis was performed by one-way analysis of variance and Bonferroni comparison post-test in **c**, and Student's *t*-test in other experiments (*P<0.05).

Rapidly dividing cancer cells require three basic outputs from metabolism: energy in the form of ATP, building blocks for macromolecular synthesis and cellular assembly and molecules to maintain the proper cellular redox environment^24^. Upregulation of the PPP flux induced by O-GlcNAcylation of G6PD would provide cells with pentose sugars for nucleotides and nucleic acid biosynthesis, as well as reducing equivalents from NADPH for lipid biosynthesis and to combat oxidative stress. We compared DNA synthesis in WT G6PD or S84V G6PD replacement A549 cells under hypoxic conditions using the 5-bromo-2'-deoxyuridine incorporation assay. DNA synthesis was significantly increased in WT G6PD replacement A549 cells compared with the S84V mutant replacement cells (Fig. 4a). As a control, depletion of G6PD with shRNA led to a sharp inhibition of DNA synthesis. These results are consistent with the previous observation that hypoxia activates G6PD glycosylation, which leads to enhanced flux through the PPP for nucleic acid biosynthesis. Control experiments were carried out under normoxia (Supplementary Fig. 8). In addition, we found that S84V mutant replacement cells showed decreased lipogenesis compared with WT replacement cells under hypoxic conditions (Fig. 4b). Consistently, WT G6PD replacement cells exhibited significantly higher cell proliferation rate than S84V mutant replacement cells under both hypoxic and normoxic conditions, yet with more pronounced effect under hypoxia (Fig. 4c, Supplementary Fig. 9). As a control, depletion of G6PD in A549 cells inhibited cell proliferation. Addition of Nuc (four ribonucleotides and four deoxyribonucleotides) to the culture medium partially rescued cell proliferation defect in S84V G6PD replacement cells (Fig. 4c, Supplementary Fig. 9). Thus, these results suggest that O-GlcNAcylation of G6PD enhances cellular biosynthesis and promotes cell proliferation.

Consistent with increased glucose flux through the PPP, enhancing O-GlcNAc levels by OGT overexpression in WT G6PD replacement cells resulted in 1.8- and 2.6-fold increase in NADPH and reduced glutathione (GSH) levels, respectively (Fig. 4d,e). Blocking glycosylation of G6PD significantly suppressed the induction of NADPH and GSH, demonstrating the importance of O-GlcNAc glycosylation at S84 in regulating NADPH homoeostasis. To further confirm that glycosylation of G6PD plays an important role in antioxidant defense, we measured the sensitivity of A549 replacement cells to ROS production and ROS-mediated cell death. Induction of ROS levels by diamide was significantly suppressed in WT G6PD replacement cells as compared with S84V mutant replacement cells (Fig. 4f). ROS levels were significantly induced by hypoxic treatments in a larger extent in S84V mutant replacement cells (Supplementary Fig. 10). Consistently, compared with WT replacement cells, S84V mutant replacement cells were markedly more sensitive to hydrogen peroxide (H_2_O_2_) treatment, and exhibited higher percentage of ROS-induced cell death (Fig. 4g). Addition of GSH, the ROS scavenger, to the culture medium partially rescued cell proliferation defect in S84V G6PD replacement cells (Fig. 4c, Supplementary Fig. 9). Combination of GSH and Nuc appeared to augment the rescue effect. These results indicate that G6PD O-GlcNAcylation plays an important role in regulating redox homoeostasis to promote cancer cell survival and cell proliferation.

### G6PD glycosylation promotes tumor formation

To gain a better understanding of the impact of G6PD glycosylation on cancer development, we first examined whether G6PD is glycosylated in different human solid tumor cells, including breast cancer cell line, MCF7, lung cancer cell lines, H661 and A549, ovarian cancer cell line, SKOV-3, melanoma cell line, A375, and osteosarcoma cell line, U2OS (Supplementary Fig. 11). G6PD glycosylation was observed in all of these cancer cell lines even though the glycosylation levels were varied, suggesting that G6PD glycosylation is potentially linked to tumor pathology.

**Figure 5:**
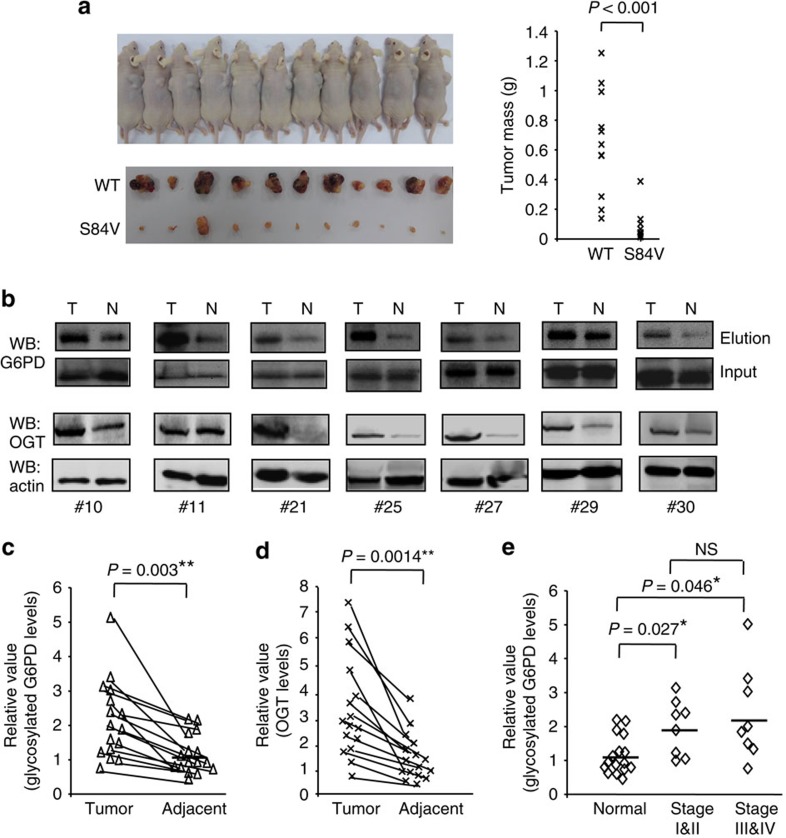
O-GlcNAcylation of G6PD is important for tumor growth *in vivo* and is increased in human lung cancers. (**a**) Tumor formation in nude mice (*n*=11) injected with WT or S84V G6PD replacement A549 cells. (Left) Dissected tumours after 7 weeks of growth in mice injected with WT replacement cells on the right flank and S84V replacement cells on the left flank. (Right) Masses of the dissected tumours. (**b**) Detection of G6PD O-GlcNAcylation and OGT expression in human lung tumor (T) tissues and the matching adjacent normal (N) tissues. (**c**–**e**) The statistical analysis of 16 paired tumor and normal samples. Relative G6PD glycosylation level was normalized to the total G6PD protein level for each patient. Statistical analysis was performed by paired Student's *t*-test (*P<0.05, **P<0.01). NS, not significant.

To determine whether G6PD glycosylation is important for tumor formation *in vivo*, we injected WT G6PD or S84V G6PD replacement A549 cells into immunocompromised mice and assayed their ability to form tumours. Mice injected with S84V G6PD replacement cells showed a significant delay in tumor formation compared with mice injected with WT G6PD replacement cells, producing tumours with much smaller total mass (Fig. 5a). Western blotting analysis confirmed that Flag-tagged WT or S84V G6PD proteins were retained in tumours and that WT G6PD was O-GlcNAcylated *in vivo* (Supplementary Fig. 12). These results are consistent with the *in vitro* cell proliferation data and demonstrate that glycosylation of G6PD at S84 provides a critical growth advantage to tumours *in vivo*.

### G6PD glycosylation is upregulated in lung cancers

Cellular O-GlcNAcylation is reported to be upregulated in various human cancers, indicating that targeting O-GlcNAcylation may serve as a novel anti-cancer strategy^25^. The finding that G6PD O-GlcNAcylation is important for A549 lung cancer cell proliferation and tumor growth prompted us to examine G6PD glycosylation in human lung cancers. We obtained a total of 39 pairs of primary human lung cancer tissue samples with matched adjacent normal lung tissues, and determined the level of glycosylation by the chemoenzymatic tagging approach and normalization to G6PD protein levels. Among these samples, eight pairs showed minimal G6PD expression in either cancer or normal tissues that prevented reliable quantification. Among the remaining 31 pairs of samples, 16 pairs showed relatively higher level of glycosylated G6PD in cancer tissues than the matched normal tissues (Fig. 5b and Supplementary Fig. 13). Quantification of these samples confirmed that the increase in the ratio of glycosylated versus total G6PD proteins is statistically significant (Fig. 5c). Previous studies showed that O-GlcNAcylation and OGT expression were significantly elevated in different cancer tissues compared with the adjacent normal tissues, including lung cancer^13^,^14^. Thus, we further analysed the expression levels of OGT protein in these samples. Among 31 pairs of samples, 20 pairs showed relatively higher level of OGT expression in cancer tissues than in the matched normal tissues (Fig. 5b,d, Supplementary Fig. 14). The samples that showed higher level of G6PD glycosylation also showed higher level of OGT expression. Thus, the increased G6PD glycosylation correlates with the increased OGT expression in lung cancer.

The development of non-small cell lung cancer is often divided into four stages (I, II, III and IV) based on tumor size and metastatic features. To determine whether G6PD glycosylation is associated with lung cancer progression, we analysed the level of glycosylation in the previous 16 paired samples according to their stages. The levels of G6PD glycosylation were increased significantly in all stages when compared with the normal tissues (Fig. 5e). However, there was no significant difference in the levels of glycosylation among different stages (Fig. 5e). Collectively, these results suggest that G6PD glycosylation may play a role in lung cancer initiation, but not progression of the disease.

## Discussion

Increasing evidence points to pivotal roles for O-GlcNAcylation in regulating a diverse set of proteins that function in transcription, insulin signalling and cellular stress response^26^. Our studies identify a previously unknown mechanism by which this modification regulates metabolic flux to promote proliferation and survival of cancer cells. Activation of G6PD activity via O-GlcNAcylation upregulates glucose flux through the PPP, leading to increased production of precursors involved in the biosynthesis of nucleotides. Enhancing flux through the PPP also provides reducing power in the form of NADPH and GSH to combat ROS, thereby supporting cell survival under oxidative stress. The dynamic induction of G6PD glycosylation in response to hypoxia and nutrient levels and coordination of metabolic reprogramming for cancer cell proliferation suggest that O-GlcNAcylation not only serves as a cellular sensor of metabolic states, but also constitutes a key metabolic regulator of glucose flux. Our study also provides a new mechanistic insight into the expanding scope of redox homoeostasis in cells.

It has long been established that the activities of metabolic enzymes are regulated by small-molecule metabolites. In contrast to the non-covalent, often transient, interactions of allosteric modulators, the O-GlcNAcylation is covalent, yet reversible and dynamically responsive to metabolic status. In addition, unlike the small molecules UDP-GlcNAc and O-GlcNAc are not primary or secondary metabolites. This may represent a difference in the function of O-GlcNAc compared with those small molecules. Given that many metabolic enzymes have been recently shown to be acetylated^27^,^28^,^29^, phosphorylated^30^,^31^ and glycosylated^17^,^23^, the post-translational modification of metabolic enzymes may serve as a general mechanism for controlling cellular metabolism.

Elevated PPP flux has been demonstrated in cancer cells^5^. In addition to providing precursors for anabolic biosynthesis and maintaining cellular redox homoeostasis, elevated PPP activity is also linked to cancer cell invasion, metastasis and resistance to cancer therapies. A plethora of mechanisms including activation of oncogenic signalling pathways and inactivation of tumor suppressors have been shown to regulate the PPP flux in a tightly controlled fashion^6^. More recent studies demonstrate that protein lysine acetylation positively or negatively regulates activities of key enzymes in the PPP, providing a selective metabolic requirement for cell proliferation and cell survival under oxidative stress^32^,^33^. Our findings that O-GlcNAcylation positively regulates G6PD activity add a new mechanistic insight into the regulation of PPP, and suggest that modulating G6PD activity may represent a potential therapeutic strategy for cancer.

## Methods

### Cell culture and tumor tissues

The cell lines 293T, A549, MCF7, H661, SKOV-3, A375 and U2OS were obtained from ATCC and cultured according to ATCC protocols. Lung tumor tissues and matching tumor-adjacent normal tissues from the same patient were obtained from the Zhejiang Cancer Hospital Bio-specimen Repository (Hangzhou, China). Informed consent was obtained from the patients. Procedures related to human subjects were approved by the Ethic Committee of Zhejiang Cancer Hospital.

### Immunoblotting

Cells were lysed in SDS lysis buffer (1% SDS, 50 mM Tris-HCl, pH 7.5, 100 mM NaCl, and Complete protease inhibitors (Roche)), and the lysate was resolved on a 4–12% SDS-PAGE gel, transferred to nitrocellulose membrane, and immunoblotted with the indicated antibodies. Antibodies used in this study were obtained from the following sources: anti-O-GlcNAc antibody (clone 18B10.C7, Thermo Scientific, 1:1,000 final dilution), anti-G6PD antibody (clone G12, Santa Cruz Biotechnology, 1:1,000), anti-Flag antibody (clone M2, Sigma-Aldrich, 1:5,000 final dilution) anti-GAPDH antibody (clone G6, Santa Cruz Biotechnology, 1:1,000 final dilution). All protein concentrations were measured using the Bicinchoninic Acid protein assay (Pierce). Western blots were visualized and quantified using an Odyssey Infrared Imaging System (LI-COR Biosciences, Version 2.1). The uncropped blots for main figures were presented in Supplementary Figs 15 and 16.

### Generation of stable cell lines

To generate the G6PD rescue A549 cell lines, Flag-tagged WT G6PD (cDNA clone obtained from Origene) and S84V G6PD were cloned into the expression vector pLenti-FlagN-shRNA^34^. This vector allows for expression of an exogenous gene with the simultaneous knockdown of the endogenous gene. To knockdown endogenous G6PD, the shRNA sequence 5′-CCGGGCTGATGAAGAGAGTGGGTTTCTCGAGAAACCCACTCTCTTCATC-AGCTTTTTG-3′ (obtained from the Public TRC Portal database) or the corresponding scramble sequence 5′-CCGGTCCTAAGGTTAAGTCGCCCTCGCTCGAGCGAGGG-CGACTTAACCTTAGGTTTTTG-3′ was inserted into the same vector. The G6PD sequence was made resistant to the G6PD shRNA by introducing silent mutations (lower case: 5′-GtTGATGAAGcGAGTGGGTTTC-3′) using the enzyme PrimeSTAR Max DNA Polymerase (Takara). Lentiviruses were produced from these constructs using a three-plasmid packaging system as described^23^. A549 cells were infected with the lentiviruses and selected for monoclonal cells with green fluorescence.

### Analysis of G6PD glycosylation

Chemoenzymatic labelling and biotinylation of proteins in cell lysates were carried out as described previously^16^ Briefly, cell lysate (500 μg) was labelled according to the Click-iT O-GlcNAc Enzymatic Labelling System protocol (Invitrogen), and conjugated with an alkyne-biotin compound as per the Click-iT Protein Analysis Detection Kit protocol (Invitrogen). Control experiments were carried out in parallel in the absence of the labelling enzyme GalT or UDP-GalNAz. Biotinylated lysates were precipitated using methanol and chloroform as described in the Click-iT Protein Analysis Detection Kit protocol, resolubilized in 1% SDS, and neutralized with an equal volume of neutralization buffer (6% NP-40, 100 mM Na_2_HPO_4_, 150 mM NaCl). Lysates were then incubated with strepavidin resin (Pierce) with end-to-end rotation at 4 °C overnight. Resin was then washed five times with 1 ml of low-salt buffer (100 mM Na_2_HPO_4_, 150 mM NaCl, 0.1% SDS, 1% Triton X-100, 0.5% sodium deoxycholate) and five times with 1 ml of high-salt buffer (100 mM Na_2_HPO_4_, 500 mM NaCl, 0.2% Triton X-100). Biotinylated proteins were eluted by boiling the resin in 50 mM Tris-HCl pH 6.8, 2.5% SDS, 100 mM DTT, 10% glycerol and 20 mM biotin for 10 min. Western blotting analysis was carried out with anti-G6PD or anti-Flag antibodies.

To quantify the level of glycosylation, the intensity of the total G6PD protein band (Input) and the glycosylated G6PD protein band (Elution) were measured, and the ratio of the intensity of the glycosylated protein versus the intensity of the total protein was taken as the level of glycosylation.

### G6PD purification and enzymatic assays of G6PD and OGT

G6PD expression constructs were individually transiently transfected into 293T cells and allowed for expression for 48 h. To purify Flag-tagged G6PD, cells were lysed in Triton X-100 lysis buffer (50 mM Tris-HCl, pH 7.4, 150 mM NaCl, 1% Triton X-100, 5 mM thimet-G and Complete protease inhibitor cocktail). The lysate (7 mg) was diluted to 2 mg ml^−1^ with NETFS buffer (100 mM NaCl, 50 mM Tris-HCl pH 7.4, 5 mM EDTA, 5 mM thiamet-G and Complete protease inhibitor cocktail). The sample was incubated with anti-Flag M2 affinity gel (400 μl; Sigma-Aldrich) at 4 °C overnight with end-to-end rotation. The gel was then washed twice with 10 ml of NETFS containing 1% Triton X-100, and twice with 10 ml of NETFS. The Flag-G6PD protein was eluted with the 3 × Flag peptide (Sigma-Aldrich) in NETFS buffer according to the manufacturer's protocol. The eluent was further purified and concentrated using an Amicon Ultra Centrifugal Filter (10 kDa molecular weight cutoff; Millipore) in a buffer containing 50 mM Tris-HCl pH 7.5, 100 mM KCl, 5 mM MgCl_2_ and 5% glycerol.

G6PD activity was measured using purified Flag-tagged G6PD in the reaction buffer containing 55 mM Tris-HCl pH 7.8, 3.3 mM MgCl_2_, 6 mM NADP, and 0.1 M G6P. The reaction mixture was incubated at 30 °C for 2 minutes before initiated by adding the G6PD enzyme (0.01 mg ml^−1^). Absorbance was recorded at 340 nm every 15 s for 10 min using a UV–vis spectrophotometer (SHIMADZU UV-2550). One unit of G6PD activity (IU) is defined as the amount of enzyme that catalyses the conversion of 1 μmol of NADP to NADPH per minute at 30 °C. Since absorbance ΔA (340 nm) of 1 μmol ml^−1^ of NADPH is 6.22 in a 1-cm light path, G6PD enzyme activity is calculated as:


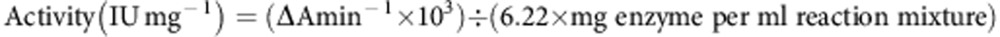


Assays to determine steady-state kinetic parameters were performed in the above-mentioned buffer with varying amounts of G6P and NADP. To ensure initial rate measurements, the amount of G6PD was adjusted to give a linear increase in readings within the first 2 min of the reaction. Kinetic parameters were calculated using the equation as described^35^.

OGT activity was determined using a modified coupled enzyme assay as described^36^ Cell lysates were concentrated to 10 mg ml^−1^ in centricon and used as OGT enzyme source. Arbitrary absorbance was normalized to total protein concentrations.

### Hypoxic and glucose treatment

Hypoxia experiments were performed in a sealed hypoxia chamber (Proox Model 110, BioSpherix, Ltd.) filled with 1% O_2_, 5% CO_2_, and 94% N_2_ at 37 °C and 60% cell confluency for the indicated periods of time. For glucose treatment experiments, A549 stable rescue cells were seeded at a density of 1 × 10^5^ cells per ml in a 10-well tissue culture plate. The cells were cultured in low glucose DMEM media (5 mM glucose) for two passages before switched to high glucose DMEM media (25 mM glucose) for the indicated periods of time.

### Glucose uptake assay

Glucose uptake assay was performed using the Glucose Uptake Fluorometric Assay Kit (BioVison) according to the manufacturer's instructions. Briefly, cells were seeded at 2,500 cells per well at a 96-well tissue culture plate. Cells were washed with phosphate-buffered saline (PBS) and incubated in serum-free DMEM medium for 2 h, and further incubated in hypoxic chambers for indicated periods of time. Cells were then quickly washed with PBS and glucose uptake was initiated by incubating in KRPH buffer (20 mM Hepes, 5 mM KH_2_ PO_4_, 1 mM MgSO_4_, 1 mM CaCl_2_, 136 mM NaCl, 4.7 mM KCl, pH 7.4) containing 10 mM 2-deoxyglucose for 20 min. Cells were then washed with PBS three times and proceeded to oxidation step to generate recordable fluorescence. Arbitrary fluorescence counts were normalized to total protein concentrations. For inhibitor treatment, cells were pretreated with 100 μM phloretin for 30 min before incubated with 2-deoxyglucose.

### Site mapping of G6PD glycosylation

Flag-tagged G6PD and HA-tagged OGT were co-transfected in 293T cells. After 48 h, Flag-tagged G6PD was isolated using anti-Flag M2 agarose beads as described above. The bound protein was eluted in a buffer containing 4% SDS and 100 mM Tris-HCl, pH 8.0. After SDS-PAGE (4–12% Bis–Tris gels) and staining with Bio-Safe Coomassie blue R250 (0.25%) Stain (Bio-Rad). The G6PD protein band was excised and manually digested in-gel with trypsin. The extracted peptides were lyophilized and reconstituted in 1 × binding buffer (Glycoprotein Isolation Kit WGA, Thermo Scientific) and incubated with WGA lectin resin (Glycoprotein Isolation Kit WGA, Thermo Scientific) at 4 °C with end-to-end rotation overnight. The resin was washed according to the manufacturer's protocol, and the bound peptides were eluted with the provided elution buffer. The eluent was further purified by reverse-phase HPLC (Agilent 1100) using a preparative reverse-phase column (Agilent Eclipse XDB-C18; 5 μm, 9.4 × 250 mm) and a gradient of 5–30% B buffer over 20 min at 4 ml min^−1^ (A buffer, 0.5% aqueous AcOH; B buffer, 100% MeCN). Fractions eluting between 5–12 min were collected, pooled, lyophilized and subjected to nanoLC-LTQ-CID/ETD-MS analysis on an LTQ-Orbitrap Velos as previously described^37^. Data search were performed by Proteome Discovery (MASCOT search engine, version 1.3) with O-GlcNAc (Ser/Thr) set as variable modification.

### Analysis of the PPP activity

PPP activity was determined by following the procedure as described^23^. Briefly, 2 × 10^6^ cells (WT or S84V G6PD A549 replacement cells, with or without OGT overexpression) were grown in a 6-cm culture plate in sodium bicarbonate-free RPMI medium supplemented with 10% FBS, 20 mM HEPES, 5 mM glucose and 0.2 μCi of [1-^14^C]-glucose or [6-^14^C]-glucose (American Radiolabeled Chemicals). The cells were placed in a closed glass vial, the centre of which was covered with filter paper soaked in 100 μl of 5% KOH, and incubated at 37 °C for 4 h. The filter paper was removed and the radioactivity was determined using a LS 6500 Multi-Purpose Scintillation Counter (Beckman Coulter). PPP activity was calculated as the difference between the radioactivity levels of samples obtained from [1-^14^C]-glucose and samples obtained from [6-^14^C]-glucose, normalized to cell number.

Determination of PPP flux by stable isotope labelling was followed as described^23^. Briefly, WT or S84V G6PD A549 replacement cells (with or without OGT overexpression) were seeded at a concentration of 200,000 cells per well in a 6-well tissue culture plate and grown in complete RPMI 1640 culture medium for 24 h. Cells were pulse labelled in RPMI 1640 supplemented with 5 mM [1,2-^13^C]-glucose and 2 mM glutamine for 4 h, and subjected to metabolite extraction with 80% aqueous methanol and dried by speedvac. Cell extracts were then analysed for relative abundance of ^13^C-metabolites by liquid chromatography-triple quadrupole mass spectrometry using scheduled selective reaction monitoring for each metabolite of interest, with the detector set to negative mode as described^23^. Extracted metabolite concentrations were calculated from standard metabolite build-up curves using natural ^12^C synthetic metabolites and normalized against cell number as well as the internal ^13^C-labelled metabolite standards added at the time of metabolite extraction. Calculations for relative percentage of PPP flux were as described^38^.

### Determination of NADPH and GSH levels

NADPH levels were determined using a colorimetric NADP^+^/NADPH Quantitation Kit (BioVision) according to the manufacturer's protocol. The signal at 450 nm was recorded using a Victor 3 microplate reader and normalized to protein concentration. GSH levels were measured using a Glutathione Assay Fluorimetric Kit (Sigma-Aldrich) according to the manufacturer's procedure. Fluorimetric signal was recorded on a Victor 3 microplate reader and normalized to protein concentration.

### LC–MS for metabolite analysis

The profiling of representative metabolites in PPP, glycolysis and tricarboxylic acid cycle were carried out on an Xevo TQ-S tandem mass spectrometer equipped with an electrospray source operating in the negative-ion multiple-reaction monitoring mode. The ion source settings were as follows: ion spray voltage, −3500 V; source temperature, 120 °C; desolvation gas, cone gas and nebulizer gas at settings 600 l h^−1^, 150 l h^−1^ and 7 Bar, respectively. Chromatographic separation was performed on a Phenomenex Luna Amide column (5 μm, 100 × 2.0 mm). The quantitative multiple-reaction monitoring transition of each particular metabolite was described as below, *m/z* 259>97 for G6P; *m/z* 259>97 for fructose-6-phosphate (F6P); *m/z* 339>97 for fructose-1,6-diphosphate; *m/z* 169>97 for dihydroxy-acetone-phosphate (DHAP) and glyceraldehyde-3-phosphate; *m/z* 185>79 for 2-phosphoglycerate and 3-phosphoglycerate (2PG/3PG); *m/z* 167>79 for phosphoenolpyruvate; *m/z* 87>43 for pyruvate; *m/z* 808>79 for acetyl-coenzyme A; *m/z* 191>111 for citrate; *m/z* 191>73 for isocitrate; *m/z* 145>101 for 2-oxoglutarate; *m/z* 117>73 for succinate; *m/z* 115>71 for fumarate; *m/z* 133>115 for malate; *m/z* 131>87 for oxaloacetate; *m/z* 173>85 for *cis*-aconitate; *m/z* 275>79 for 6-phosphogluconate; *m/z* 229>97 for ribose-5-phosphate, ribulose-5-phosphate and xylulose-5-phosphate; *m/z* 289>97 for sedoheptulose-7-phosphate; *m/z* 199>97 for erythrose-4-phosphate. Other MS/MS parameters, such as declustering potential and collision cell energy, were optimized for each particular metabolite^39^. Data were acquired and processed using MassLynx software (Version V4.1). Peak areas of individual metabolites were normalized against the total protein amount. The fold changes of the relative level of targeted metabolites are calculated (Data are presented as the mean±s.d. of triplicate biological experiments). The mass spectrometry raw data have been deposited in the iProX database.

### Measurement of lipid synthesis

Cellular lipid synthesis was measured by incorporation of ^14^C-glucose. Cells were seeded at subconfluency on a six-well tissue culture plate. Cells were labelled with 4 μCi ml^−1^ of ^14^C-glucose for 2 h, followed by washing twice with cold PBS. Lipids in cells were extracted with 800 μl of hexane:isopropanol (3:2, v/v). Extracts were dried by speedvac, resuspended in 50 μl of chloroform, and subjected to scintillation counting. Counts were normalized with cell numbers.

### Measurement of ROS levels and H_2_O_2_-mediated cell death

A redox-sensitive dye 5(6)-chloromethyl-2'7'-dichlorodihydrofluorescein diacetate-acetyl ester (CM-H_2_DCFDA; Molecular Probes) was used to measure ROS levels in cell lines as previously described^23^. Briefly, cells were cultured overnight at a density of 4 × 10^5^ cells per well in a 12-well tissue culture plate in complete DMEM media. After treating cells with various concentrations of diamide (Sigma-Aldrich) as indicated for 10 min, fresh culture media were added. The cells were then incubated with 10 μM CM-H_2_DCFDA for 20 min and rinsed three times with PBS. The cells were lysed in 1% SDS and sonicated. The mixture was centrifuged (15,000 × *g*, 2 min) to remove any cell pellets. 100-μl aliquots were taken, and the fluorescent intensity was measured on a Victor 3 microplate reader. Signal intensity was normalized to protein concentration.

The percentage of cell death was measured using a lactate dehydrogenase (LDH)-based toxicology assay kit (Sigma-Aldrich) according to the manufacturer's protocol. The percentage of cell death was determined by comparing the amount of cytoplasmic LDH released into the culture medium relative to the total cytoplasmic LDH, as determined by the reduction of NAD^+^.

### Cell proliferation analysis

Cell proliferation assays were performed by seeding 2,000 cells per well in a 96-well tissue culture plate in normoxia. Twenty-four hours after seeding, cells that were used for hypoxic treatments were further cultured in the sealed hypoxic chamber as mentioned above. Cell proliferation was determined by counting cell numbers as stained by trypan blue every 24 h for 96 h.

### Xenograft studies

Immunocompromised mice (BALB/c-nude, male, 5–6 week old, Charles River Laboratories) were injected subcutaneously with 4.5 × 10^6^ cells each (resuspended in 100 μl of serum-free F12-K medium) in the right flank (WT G6PD A549 replacement cells) and the left flank (S84V G6PD A549 replacement cells). Tumor growth was monitored every 3 days over a 7-week period. At the end of the seventh week, the tumours were harvested and weighed. Experiments were performed in accordance with the Zhejiang University Institutional Animal Care and Use Committee guidelines.

### Statistical analysis

*P* values were calculated from Student's paired *t*-test when comparing within groups and from Student's unpaired *t*-test when comparing between groups. For those analyses where more than one *t*-test is applied to the same data set, statistical analysis was performed by one-way analysis of variance and Bonferroni comparison post-test.

## Additional information

**Accession code:** The mass spectrometry data have been deposited in the iProx database under accession code IPX00037500.

**How to cite this article:** Rao, X. *et al.* O-GlcNAcylation of G6PD promotes the pentose phosphate pathway and tumor growth. *Nat. Commun.* 6:8468 doi: 10.1038/ncomms9468 (2015).

## Supplementary Material

Supplementary Information Supplementary Figures 1-16 and Supplementary Tables 1-2
