# Supplementary material for: O-GlcNAcylation of G6PD promotes the pentose phosphate pathway and tumor growth
Source: Nat Commun. 2015 Sep 24;6:8468. doi: 10.1038/ncomms9468 (PMC4598839; doi:10.1038/ncomms9468)
Supplement: Supplementary Information — Supplementary Figures 1-16 and Supplementary Tables 1-2 [file ncomms9468-s1.pdf]

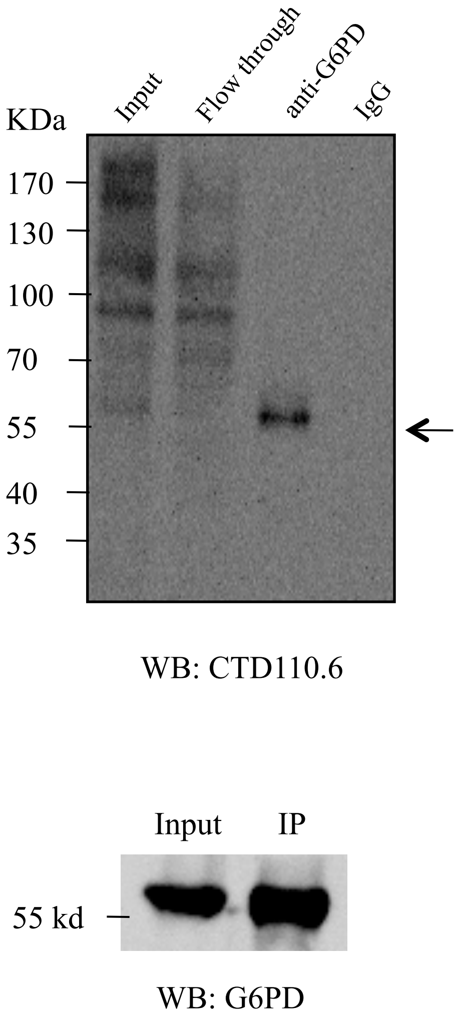

Supplementary Figure 1. Detection of O-GlcNAcylation on G6PD with a pan-anti-O-GlcNAc antibody (CTD110.6). Endogenous G6PD was immunoprecipitated from cell lysates and immunoblotted with CTD110.6 and G6PD antibody.

A

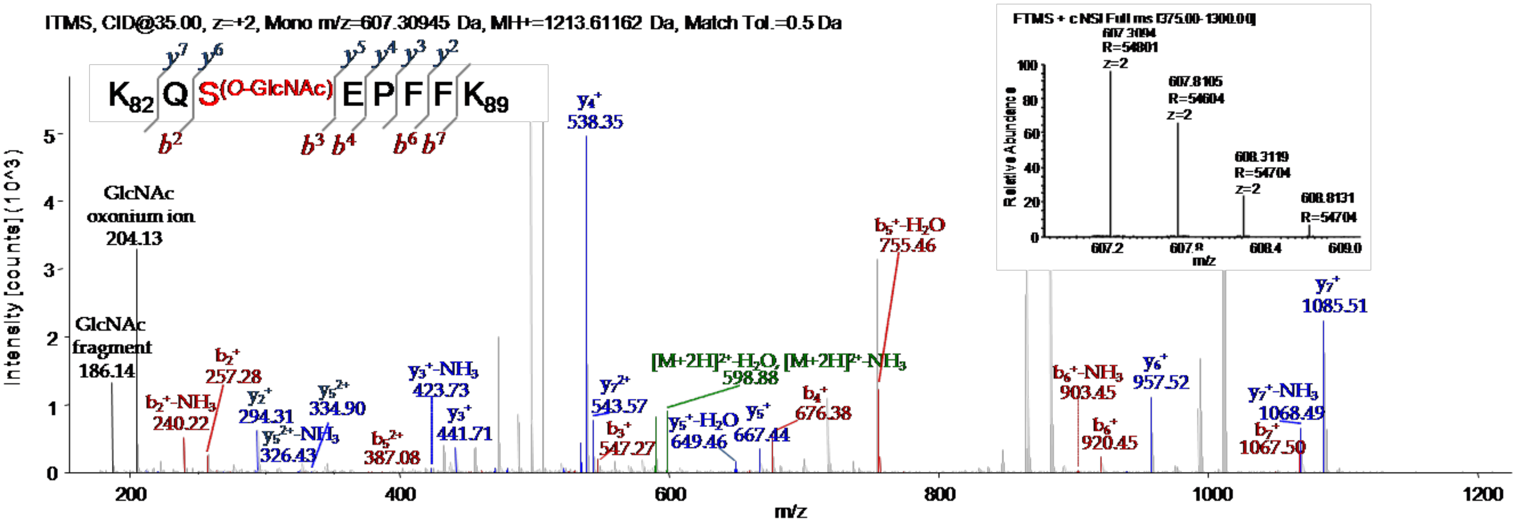

B

Homo sapiens	RLTVADIRKQSEPFFKATP	92
Mus musculus	RLTVDDIRKQSEPFFKATP	92
Ovis aries	RLTVADIRKQSEPFFKATP	92
Salmo salar	DLTVDAIKTASMPYMKVAD	96
Macropu robustus	NLTVDDIRKQSEPYFKATP	92

Supplementary Figure 2. G6PD is *O*-GlcNAc glycosylated at Ser84. (**A**) Mapping *O*-GlcNAc glycosylation site on G6PD using mass spectrometry. Flag-tagged human G6PD was expressed in 293T cells co-transfected with OGT, isolated using anti-Flag M2 affinity resin, and digested with trypsin. The *O*-GlcNAc-modified peptides were subjected to LTQ-Orbitrap MS/MS analysis. The b and y fragment ions observed were used to map the glycosylation site to the serine residue indicated in red. (**B**) Sequence alignment of the residues surrounding Ser84 of G6PD across different species.

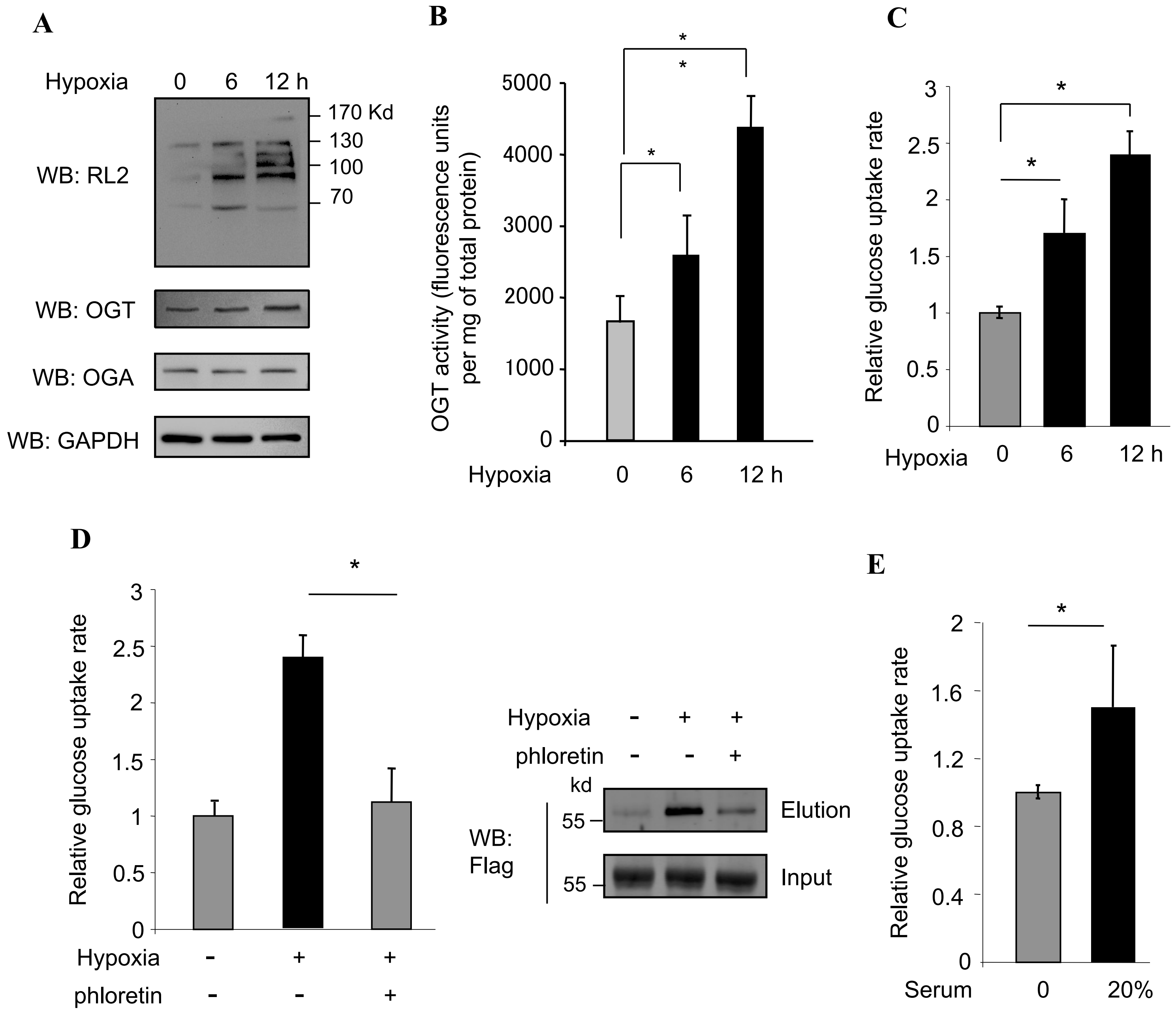

Supplementary Figure 3. (**A**) Cellular O-GlcNAcylation levels, endogenous OGT and OGA expressions upon hypoxic treatments. (B) OGT activity under hypoxic treatments (n =3 assays). (C) Relative glucose uptake rate in A549 cells expressing Flag-tagged G6PD upon hypoxic treatments for the indicated periods of time (n = 3 experiments). (D) Relative glucose uptake rate and G6PD O-GlcNAcylation levels in A549 cells expressing Flag-G6PD under hypoxia upon the treatment of glucose uptake inhibitor (n = 3 assays). (E) Relative glucose uptake rate in A549 cells expressing Flag-tagged G6PD upon serum stimulation (n = 3 assays). Error bars denote the standard deviation of the mean (mean ± SD). Statistical analysis was performed by one-way analysis of variance (ANOVA) and Bonferroni comparison post-test in (B and C), and Student's *t* test in (D and E)(*$P < 0.05$).

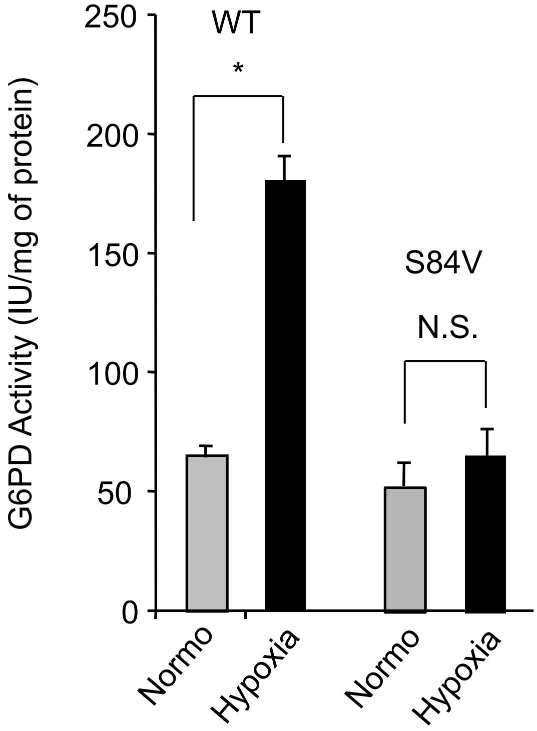

Supplementary Figure 4. Enzymatic activities of WT and S84V G6PD purified from 293T cells (n = 3 experiments) under normoxia and hypoxia treatments. Error bars denote the standard deviation of the mean (mean ± SD). Statistical analysis was performed by Student's t-test. (*P < 0.05, N.S. Not Significant)

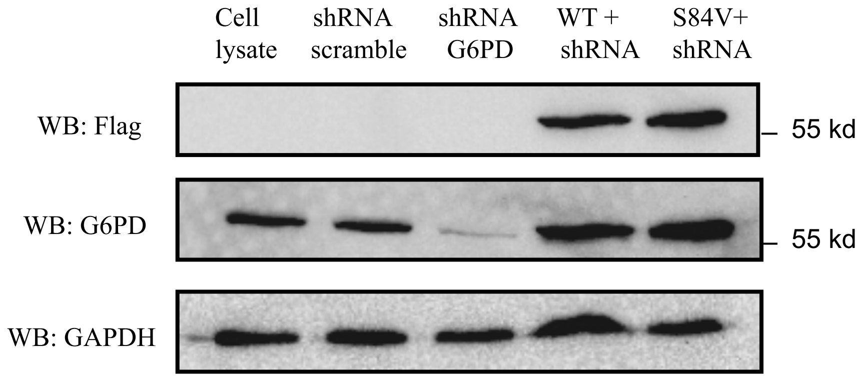

Supplementary Figure 5. Generation of WT G6PD and S84V G6PD rescue A549 cell lines. Endogenous G6PD was depleted with G6PD shRNA. shRNA-resistant Flag-tagged WT or S84V G6PD was stably expressed in A549 lung cancer cells.

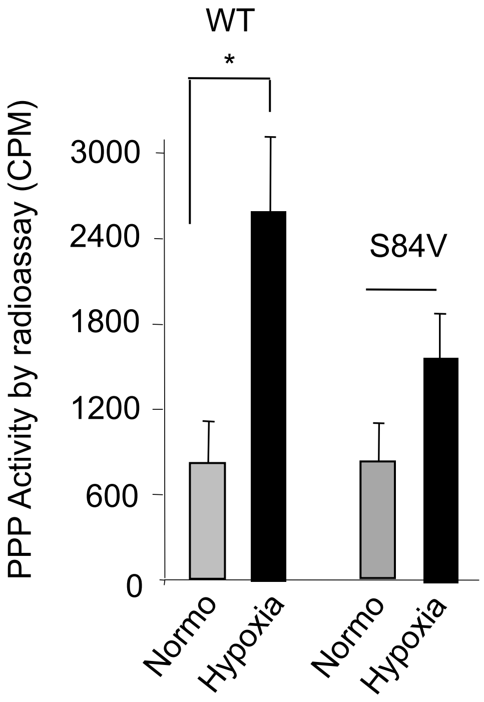

Supplementary Figure 6. PPP flux activity in WT and S84V G6PD replacement cells under normoxia and hypoxia treatments, as determined by radioassays. Error bars denote the standard deviation of the mean (mean ± SD). Statistical analysis was performed by Student's t-test. (*P < 0.05)

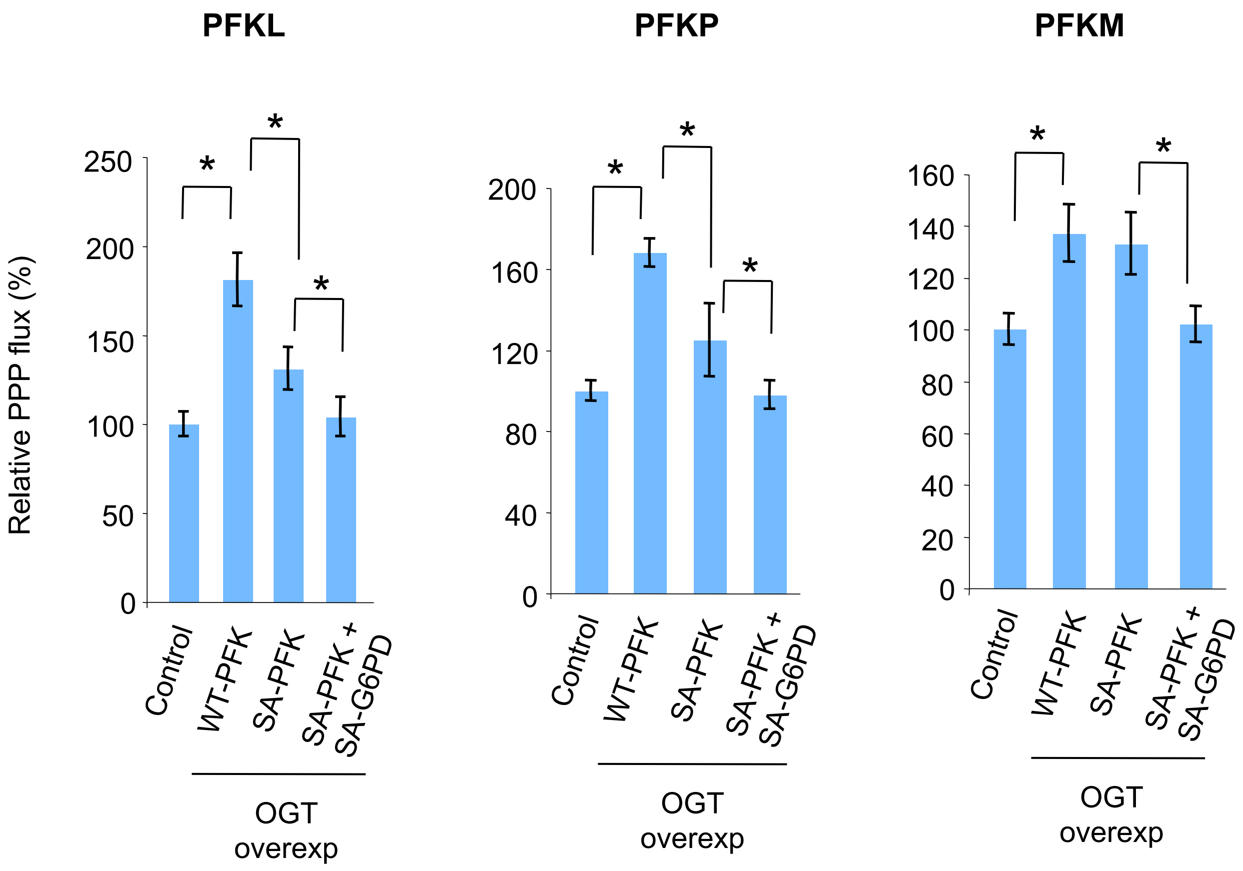

Supplementary Figure 7. Investigation of the differential contribution of PPP flux by O-GlcNAcylation of PFK1 isoforms and G6PD. The error bars represent mean values ±SD from three replicates (*, p<0.05), n = 3 experiments. Statistical analysis was performed by one-way analysis of variance (ANOVA) and Bonferroni comparison post-test.

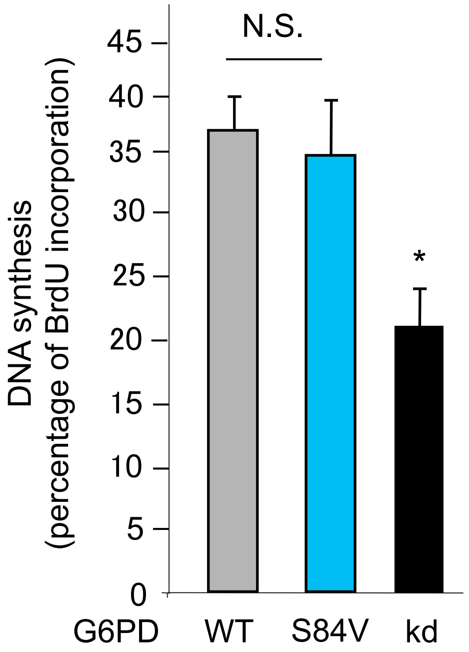

Supplementary Figure 8. DNA synthesis in WT and S84V G6PD replacement A549 cells under normoxia conditions, as determined by BrdU incorporation assays. Control experiment was performed in G6PD depleted A549 cells (n = 3 assays). . Error bars denote the standard deviation of the mean (mean ± SD). Statistical analysis was performed by Student's t-test. (* $P < 0.05$, N.S. Not Significant)

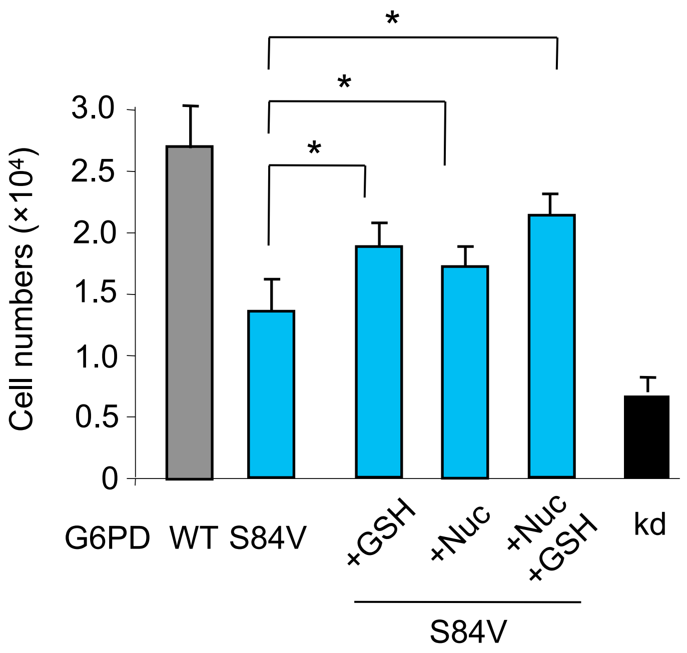

Supplementary Figure 9. Cell proliferation rate of G6PD WT and S84V replacement A549 cells under normoxic conditions. Cell numbers were measured by trypan blue counting 4 days after the initial seeding. The error bars represent mean values ±SD from three replicates (*, $p<0.05$). N = 3 experiments. Statistical analysis was performed by one-way analysis of variance (ANOVA) and Bonferroni comparison post-test.

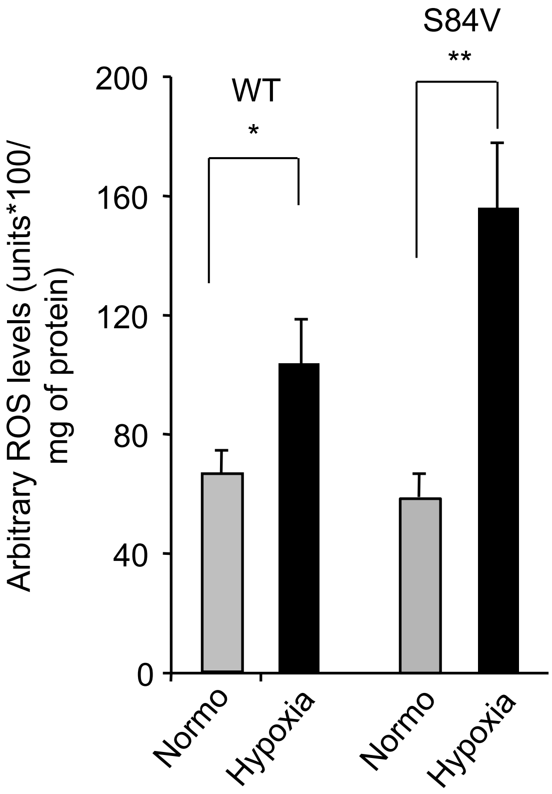

Supplementary Figure 10. ROS levels in WT and S84V G6PD replacement cells under normoxia and hypoxia treatments. The error bars represent mean values ±SD from three replicates (*, $p<0.05$). N = 3 experiments. Statistical analysis was performed by Student's t-test.

A

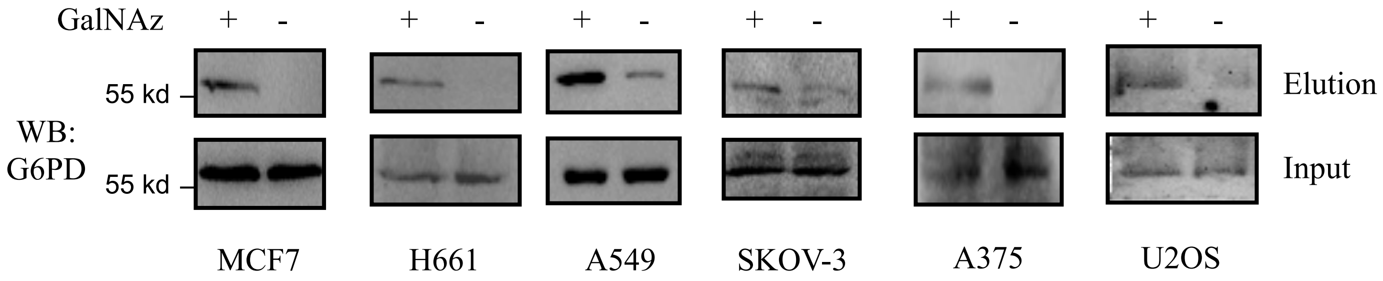

B

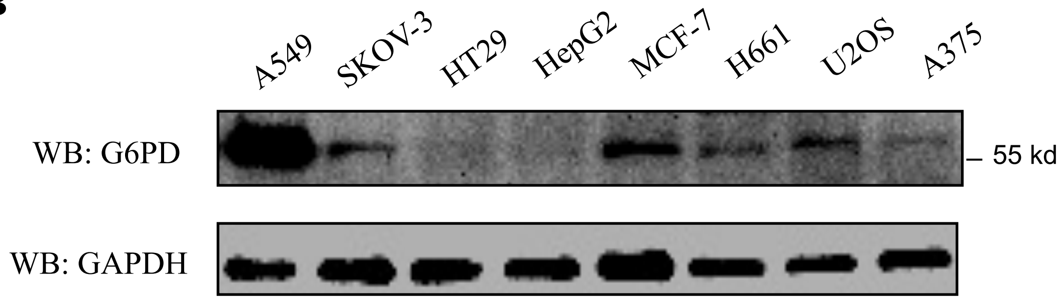

Supplementary Figure 11. (A) Detection of G6PD glycosylation in various human solid tumor cell lines. (B) Detection of endogenous G6PD expression in various solid tumor cell lines.

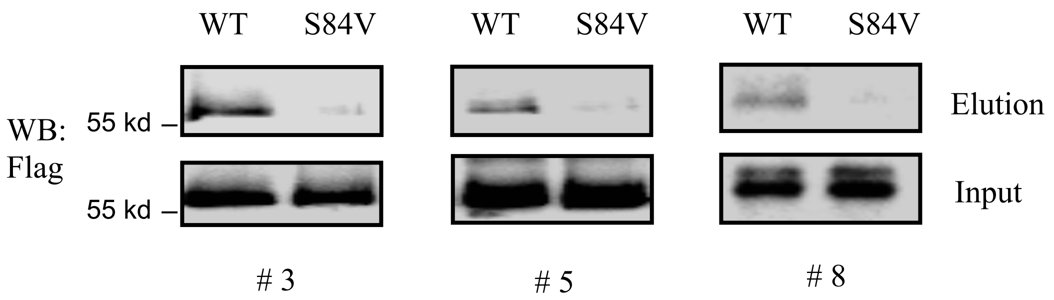

Supplementary Figure 12. Detection of G6PD glycosylation in three representative mouse tumors originating from WT or S84V rescue A549 cells.

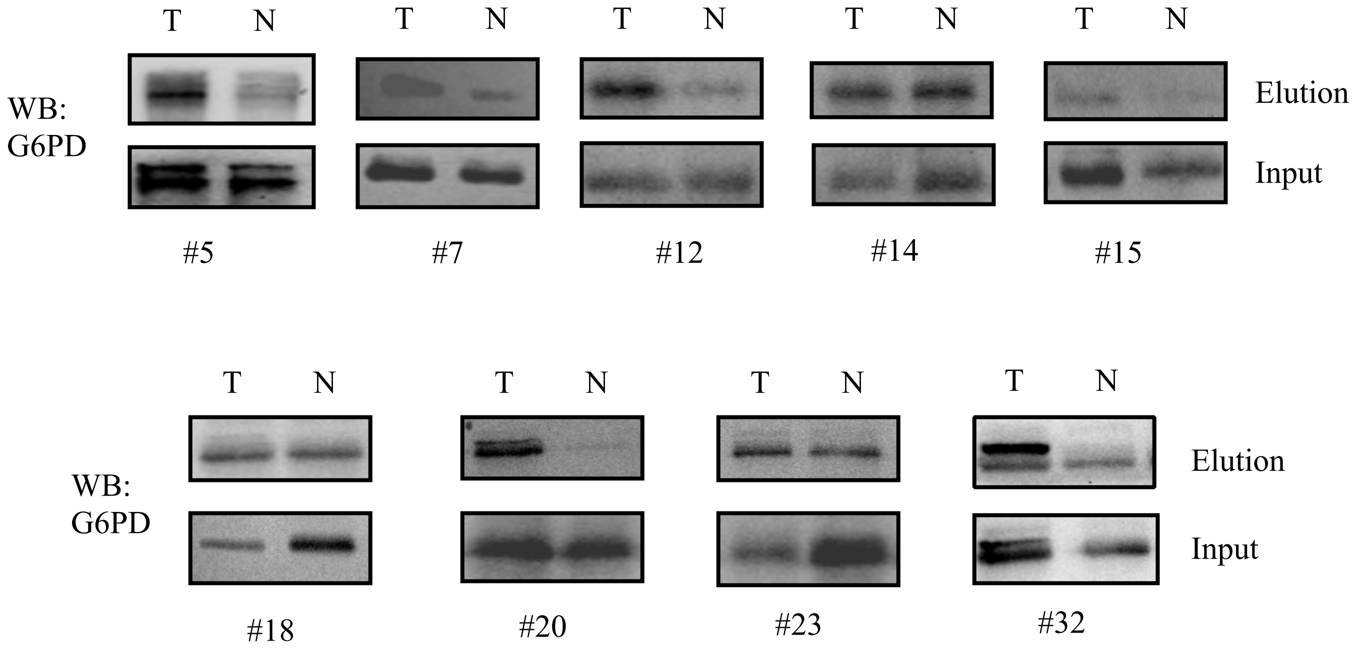

Supplementary Figure 13. Detection of G6PD O-GlcNAcylation in 16 pairs of human lung tumor (T) tissues and the matching adjacent normal (N) tissues. The data for the rest 7 pairs of samples were shown in the main text figures.

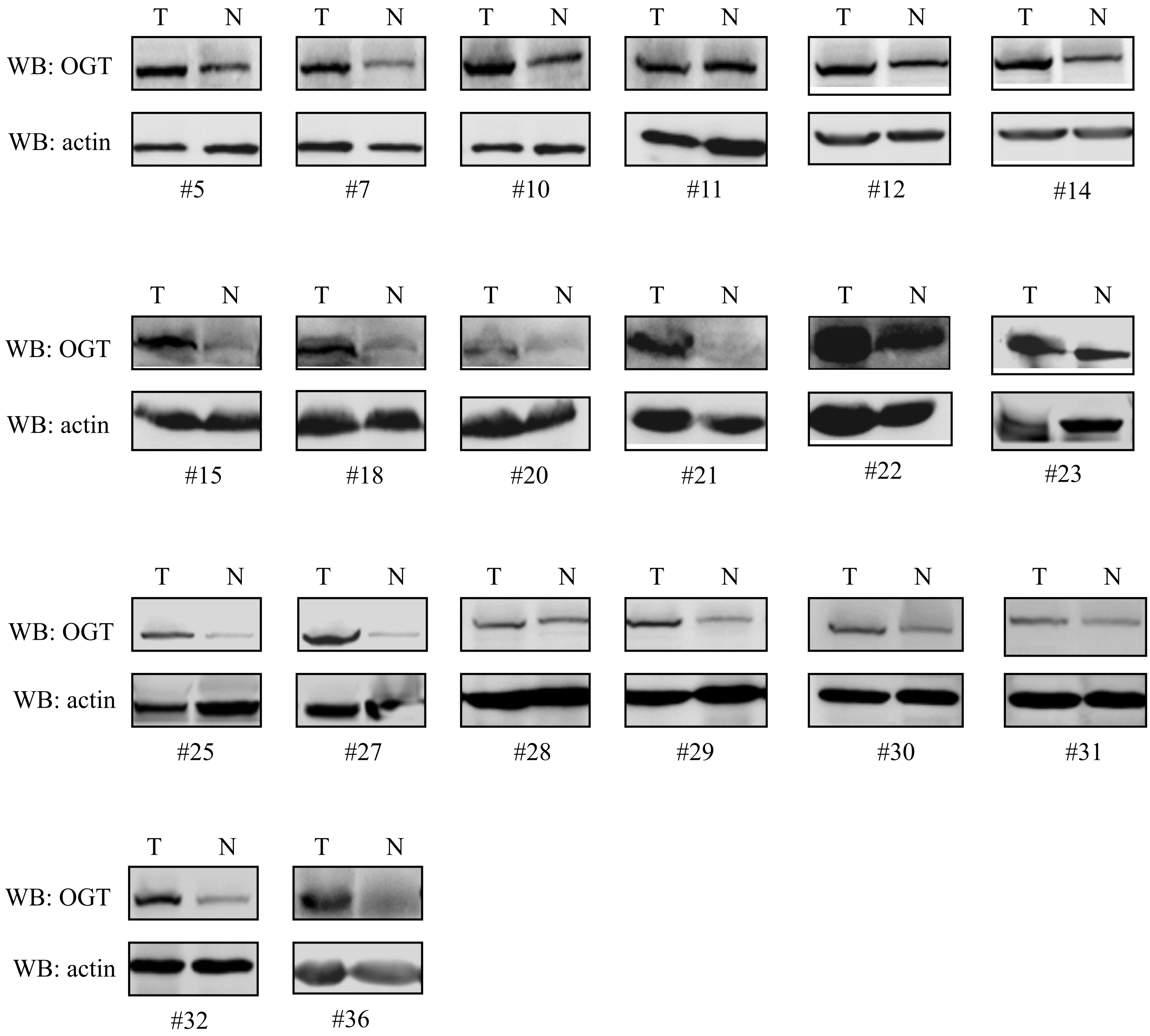

Supplementary Figure 14. Detection of OGT expression in 20 pairs of human lung tumor (T) tissues and the matching adjacent normal (N) tissues.

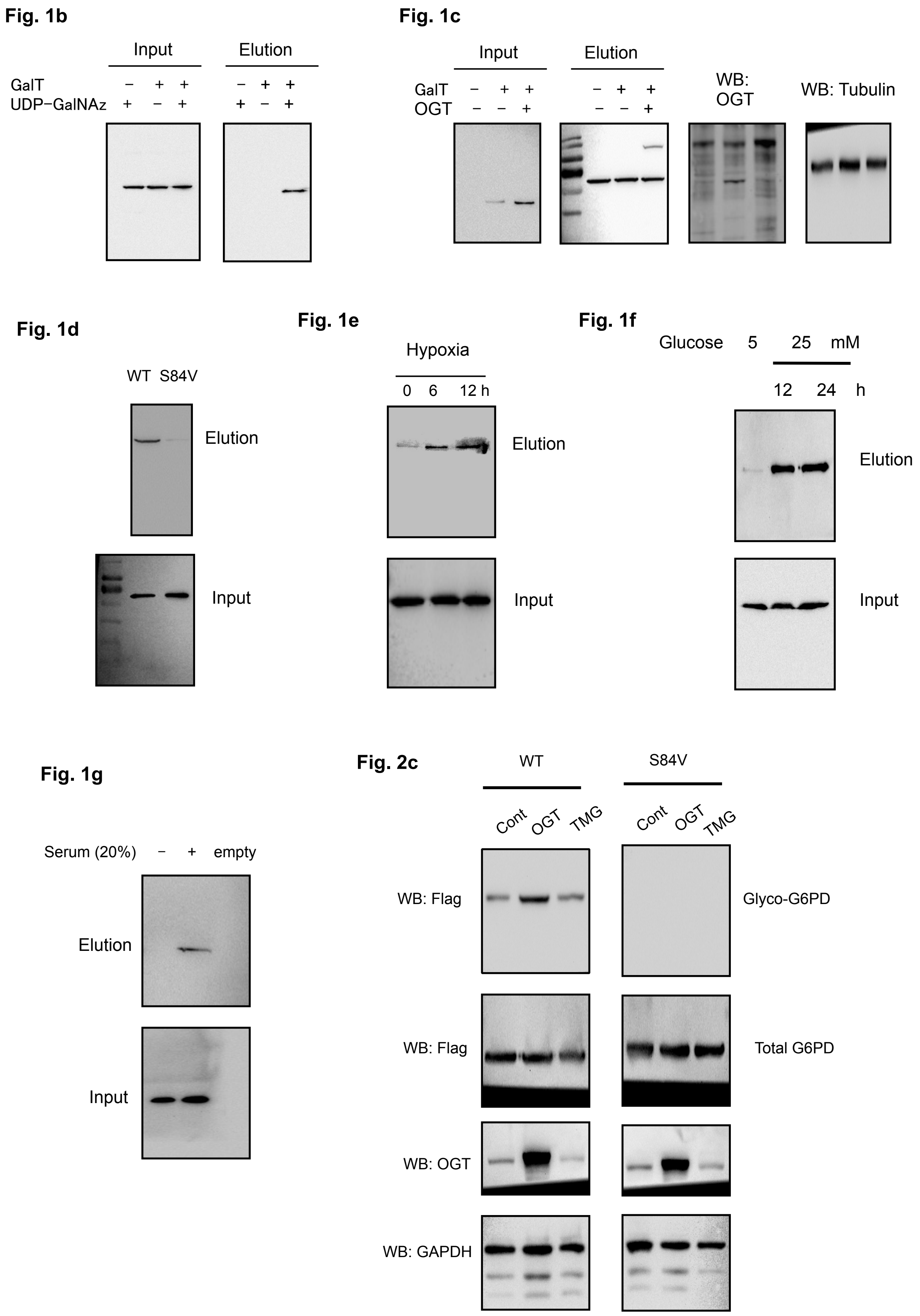

Supplementary Figure 15. Full gel blots for the figures in the main text.

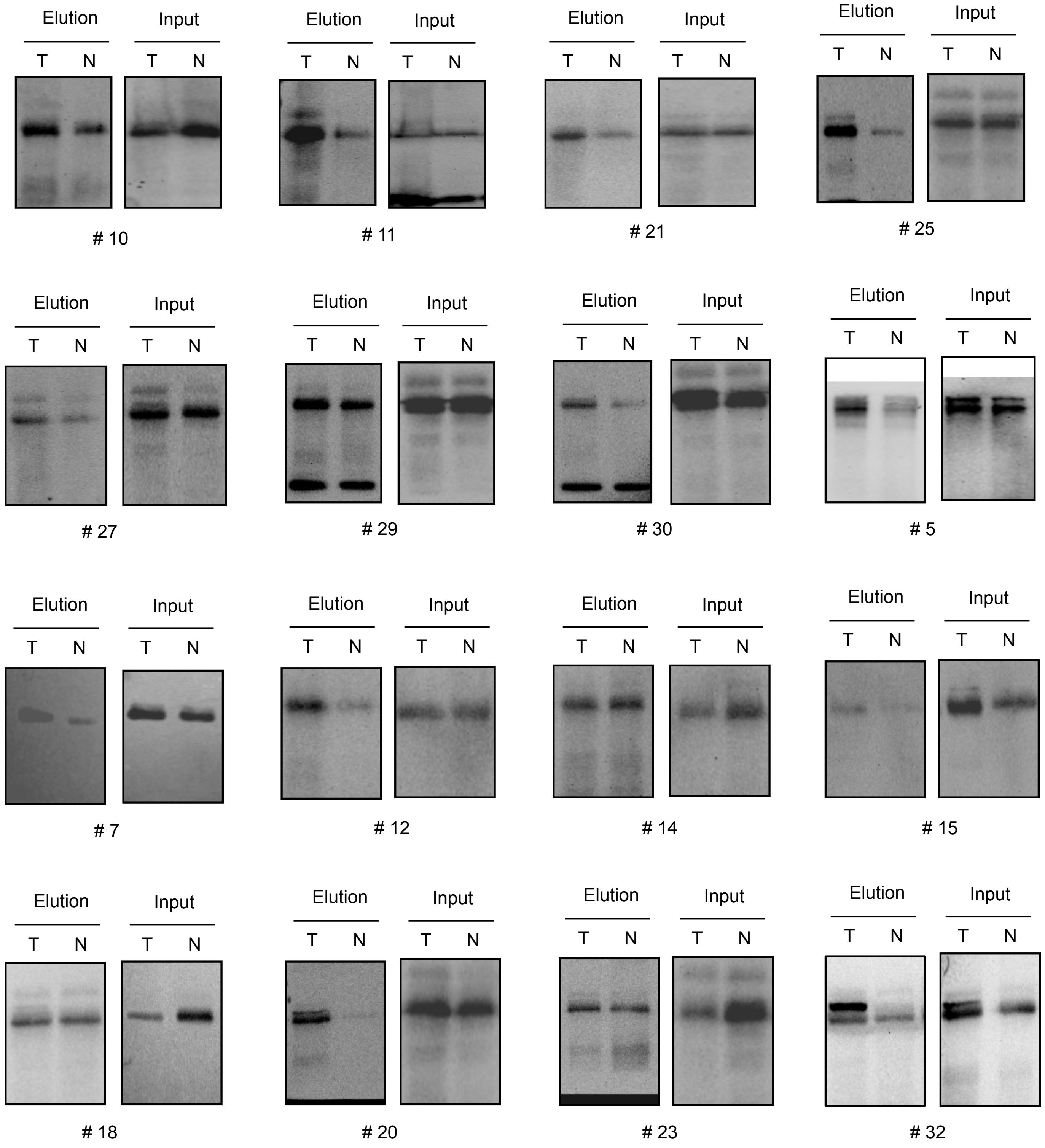

Supplementary Figure 16. Full gel blots for the detection of G6PD glycosylation in lung tumor and normal tissues.

G6PD	k_{cat} (s^{-1})	$K_{m\ G6P}$ (µM)	$K_{m\ NADP+}$ (µM)	k_{cat}/ $K_{m\ G6P}$	k_{cat}/ $K_{m\ NADP+}$	k_d (NADP+)
Low glyco	254±11	8.3±0.6	65±5	30.60±2.6	3.91±0.5	7.3±0.7
High glyco	307±9	7.2±0.4	27±3	42.64±1.3	11.37±0.9	2.6±0.5

Supplementary Table 1. Comparison of enzyme kinetics of WT G6PD expressed in 293T cells in the absence or presence of OGT overexpression. The error bars represent mean values ±SD from three replicates. N = 3 experiments.

**Metabolites**	**WT G6PD overexp. v.s no overexp.**	
	Fold change	P value
glucose-6-P	1.25	0.41
6-P gluconate	5.12	2.3×10^{-4}
ribose-5-P	3.37	4.7×10^{-5}
deoxyribose	2.41	3.9×10^{-6}
erythrose-4-P	1.16	0.62
Sedoheptulose-7-P	0.89	1.2×10^{-3}
Fructose-1,6-BP	1.46	0.03
Glyceraldehyde-3-P	1.07	0.59
3-phosphoglycerate	1.34	0.66
phosphoenolpyruvate	0.97	0.92
pyruvate	1.18	0.27
fumarate	3.17	6.3×10^{-4}
malate	2.17	2.6×10^{-3}
Alpha-ketoglutarate	0.52	1.9×10^{-5}
aconitate	1.49	0.82
oxaloacetate	1.55	0.11

Supplementary Table 2. Targeted analysis of abundance of different metabolites in major glucose metabolic pathways in A549 cells upon G6PD overexpression (n = 3 experiments). All P values are multiple-hypothesis-corrected.
